# Tai Chi as a Therapy of Traditional Chinese Medicine on Reducing Blood Pressure: A Systematic Review of Randomized Controlled Trials

**DOI:** 10.1155/2021/4094325

**Published:** 2021-09-04

**Authors:** Xiandu Pan, Li Tian, Fan Yang, Jiahao Sun, Xinye Li, Na An, Yanfen Xing, Xin Su, Xu Liu, Can Liu, Yonghong Gao, Yanwei Xing

**Affiliations:** ^1^Beijing University of Chinese Medicine, Beijing, China; ^2^Guang'anmen Hospital, Chinese Academy of Chinese Medical Sciences, Beijing, China; ^3^Key Laboratory of Chinese Internal Medicine of the Ministry of Education, Dongzhimen Hospital Affiliated to Beijing University of Chinese Medicine, Beijing, China; ^4^Shanxi University of Chinese Medicine, Jinzhong 030619, China

## Abstract

**Objective:**

This study systematically evaluated the effects of Tai Chi exercise on blood pressure, body mass index (BMI), and quality of life (QOL) in patients with hypertension. A meta-analysis was performed to provide a reliable reference for clinical practice.

**Methods:**

We searched for randomized controlled trials (RCTs) in five English databases and two Chinese databases, with the earliest data dated December 5, 2020. A quality assessment of the methods and a meta-analysis were also conducted.

**Results:**

The meta-analysis of 24 studies showed that the intervention group showed better outcomes in terms of systolic blood pressure (SBP) (SMD −1.05, 95% CI −1.44 to −0.67, *P* ≤ 0.001; *I*^*2*^ = 93.7%), diastolic blood pressure (DBP) (SMD −0.91, 95% CI −1.24 to −0.58, *P* ≤ 0.001; *I*^*2*^ = 91.9%), and QOL (physical functioning (SMD 0.86, 95% CI 0.36 to 1.37, *P*=0.001; *I*^*2*^ = 91.3%), role-physical (SMD 0.86, 95% CI 0.61 to 1.11, *P* ≤ 0.001; *I*^*2*^ = 65%), general health (SMD 0.75, 95% CI 0.32 to 1.17, *P*=0.001; *I*^*2*^ = 88.1%), bodily pain (SMD 0.65, 95% CI 0.29 to 1.00, *P* ≤ 0.001; *I*^*2*^ = 83.1%), vitality (SMD 0.71, 95% CI 0.34 to 1.07, *P* ≤ 0.001; *I*^*2*^ = 84.3%), social functioning (SMD 0.63, 95% CI 0.07 to 1.19, *P*=0.027; *I*^*2*^ = 93.1%), role-emotional (SMD 0.64, 95% CI 0.22 to 1.06, *P*=0.003; *I*^*2*^ = 88.1%), and mental health (SMD 0.73, 95% CI 0.31 to 1.16, *P*=0.001; *I*^*2*^ = 88.2%)) compared to those of the control group. However, no significant improvements were seen in BMI of the intervention group (SMD −0.08, 95% CI −0.35 to −0.19, *P*=0.554; *I*^*2*^ = 69.4%) compared to that of the control group.

**Conclusion:**

Tai Chi is an effective intervention to improve SBP and DBP in patients with essential hypertension.

## 1. Introduction

Elevated blood pressure is the leading contributor to the global burden of disease and mortality, causing 10.7 million deaths annually [1, 2]. Globally, about a quarter of adults have hypertension, with 874 million adults having SBP greater than or equal to 140 mmHg [1]. For different reasons, chronic peripheral arteriole spasm for a long time leads to a rise in blood pressure. Persistent hypertension can change the structure of the systemic arterioles and cause pathological changes in the aorta, heart, kidney, and brain. Pharmacotherapy is mainly used in clinical settings, but this is not recommended for prehypertension patients. Therefore, nondrug treatments of hypertension are worth discussing. Like diet, lifestyle, health education, and other factors, exercise intervention can also appositively affect the treatment of hypertension. Tai Chi is a gentle, safe, and uncomplicated comprehensive exercise, which encompasses the concept of organic wholeness in traditional Chinese medicine and can help regulate body homeostasis and build up a good physique [3]. There have been a number of studies displaying the improvement of Tai Chi in eliciting SBP and DBP reductions [4–23]. Moreover, numerous health benefits of Tai Chi have been found; it may ameliorate symptoms, enhance the QOL, regulate physiological mechanisms, and postpone the senility [24–27].

The existing meta-analyses of studies on Tai Chi intervention for hypertension are of low quality since the included literature had relatively short durations of Tai Chi intervention, and none of the meta-analyses included RCTs. In addition, the number of RCTs on Tai Chi intervention for hypertension has increased over the past few years, with two even showing negative results. Therefore, we conducted a systematic review and meta-analysis of RCTs involving Tai Chi as an intervention for hypertension.

## 2. Materials and Methods

### 2.1. Eligibility Criteria

There were several criteria for inclusion: (1) Tai Chi or Tai Chi with antihypertensive drugs (AHD) or health education (HE) were used as interventions in RCT; (2) participants with primary hypertension defined according to the 2010 Chinese guidelines for the management of hypertension [28] and the 1999 WHO-International Society of Hypertension Guidelines for the Management of Hypertension [29] were included, and the selection criteria were independent of gender, race, or age-based limitations; (3) the results of the trial contained two types of data: SBP and DBP; and (4) languages were limited to Chinese, Korean, and English.

### 2.2. Exclusion Criteria

The following studies were excluded from the analysis: (1) studies with Tai Chi intervention time of fewer than three months, (2) studies with intervention measures involving traditional Chinese medicine, (3) studies that were literature reviews, and (4) studies with incorrect, incomplete, or invalid data.

### 2.3. Outcomes

The primary outcomes included SBP and DBP, while BMI and QOL were the secondary outcomes.

### 2.4. Search Strategy

With a time limit of December 5, 2020, we searched for RCTs in five English and two Chinese databases (Web of Science, PubMed, Korea Citation Index, EMBASE, the Cochrane Library, China National Knowledge Infrastructure, and Wanfang Data). We searched for terms related to Tai Chi and hypertension, namely, “Tai Ji” OR “Tai Chi” OR “Chi, Tai” OR “Tai Chi Chuan” OR “Ji Quan, Tai” OR “Quan, Tai Ji” OR “Taiji” OR “Taijiquan” OR “Taiji” OR “Tai Ji Quan,” and “Hypertension” OR “high blood pressure” OR “Blood Pressure, High” OR “blood pressure.”

### 2.5. Study Selection and Data Extraction

Two investigators ( XDP and LT) independently screened the literature and extracted studies based on the inclusion and exclusion criteria. Disagreements were resolved through negotiations. The collected information included the following: (1) basic data of the study (title, author, and publication date); (2) basic information of patients, such as age and number of patients included, (3) intervention measures of the treatment and control groups, (4) outcome index data, and (5) possible bias.

### 2.6. Quality Assessment and Publication Bias

In line with the Cochrane Handbook for Systematic Reviews of Interventions Version 6.1 [30], two investigators independently evaluated the included studies for risk of bias and distinguished their risk levels, including selection bias, performance bias, detection bias, attrition bias, reporting bias, and other biases. For publication bias, the funnel plot test and Egger's regression test were used for evaluation.

### 2.7. Statistical Analysis

The data were sorted and summarized using Excel, and Stata 16.0 2019 was used for data processing, such as heterogeneity testing, data merging, forest map, and funnel plot creation. The statistics of continuous variables are presented as standardized mean differences (SMD) and 95% confidence intervals (CI). Heterogeneity was tested using a chi-squared test and *I*^2^ statistic. In a Cochrane systematic review, as long as *I*^2^ is less than 50%, its heterogeneity is acceptable, and a fixed effects model should be selected for meta-analysis. Meanwhile, a randomized effect model should be utilized when heterogeneity between studies is significant. Subgroup analysis, meta-regression, and influence analysis were used to manage heterogeneity. If the source of heterogeneity was not found in the subgroup and meta-regression analyses, influence analysis was conducted.

## 3. Results

### 3.1. Search Strategy and Study Characteristics

**Figure 1:**
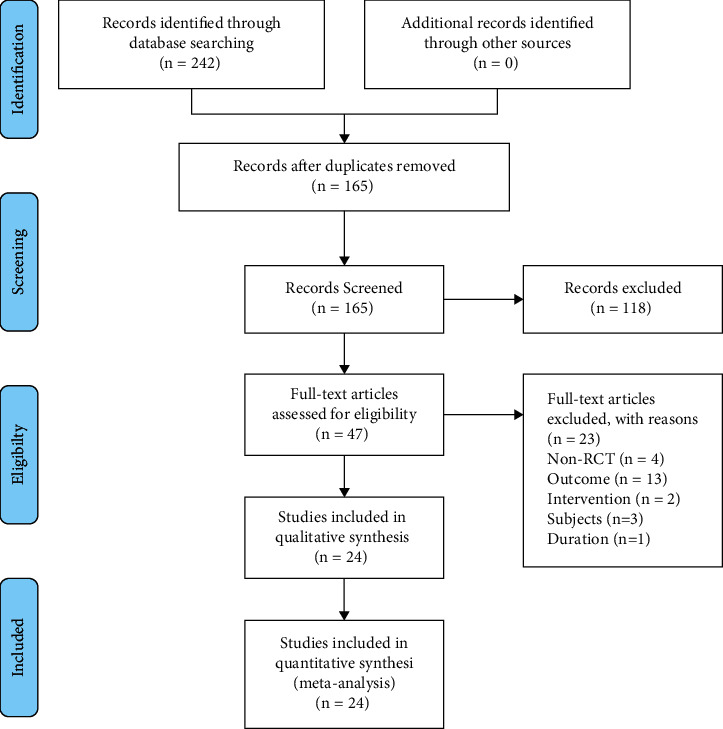
Flow diagram of the study selection process.

We identified 242 studies using the predefined search terms. In addition, 77 duplicated studies were eliminated. Based on the exclusion criteria, we excluded 118 irrelevant studies and obtained 47 potentially qualified studies upon scanning the title and abstract, 23 of which were eventually excluded when the full texts were read. Ultimately, we obtained 24 relevant RCTs for this study (Figure 1).

Overall, the 24 RCTs [4–27] included 2,095 patients, with 1,074 in the treatment group and 1,021 in the control group, as given in Table 1 and Supplementary Table 1. The intervention design included Tai Chi exercise alone or with the intake of AHD and HE as interventions. Meanwhile, the control group was no treatment in twelve studies. In three studies, the control groups performed moderate aerobic exercise (AE) without Tai Chi. For both the intervention and control groups, four studies conducted HE for patients. Regular AHD was prescribed in five studies in both the intervention and control groups. Each study intervention period ranged from 3 months to 5 years, and the outcome indicators included SBP and DBP (mmHg).

### 3.2. Quality Assessment and Publication Bias

**Figure 2:**
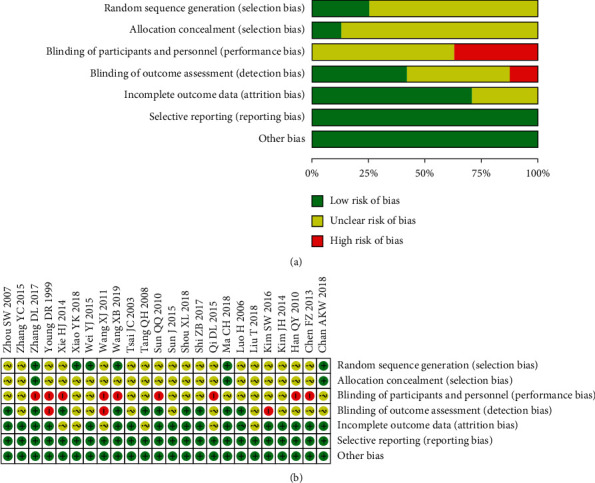
Risk of bias graph. (a) The review authors' judgements about each risk of bias item are presented as percentages across all included studies. The summaries of the risk of bias are included. (b) Review authors' judgements about each risk of bias item for each included study.

As shown in Figure 2, six studies [4, 5, 7, 11, 25, 27] described the generation of random sequences and were considered to be of low risk. The remaining studies did not describe the generation of random sequences in detail and were judged to have an unclear risk. Three studies [5, 7, 25] reported allocation concealment and were determined to be of low risk, while the studies that did not report this were considered to have unclear risks. None of the studies blinded the participants and were therefore considered to have a high risk. Two studies [7, 10] reported blinding the outcome evaluators and were judged to be at low risk. None of the participants in ten studies [4, 6, 8, 11–13, 15, 18, 20, 23] were dropped out. In seven of these studies [5, 7, 10, 17, 22, 25, 26] with dropouts, the numbers and reasons for dropping out were provided and were considered to have a low risk. The remaining studies did not mention withdrawal and were therefore considered to be at high risk. All studies reported their stated outcomes and were judged to have a low risk. We found no other bias.

**Figure 3:**
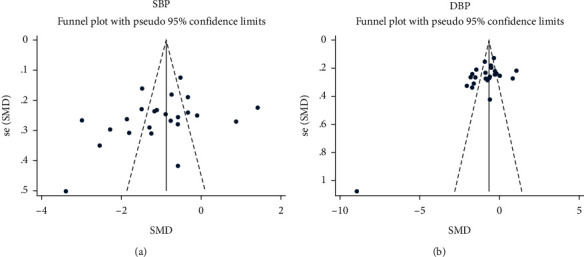
Funnel plot of studies comparing SBP (a) and DBP (b) of the intervention group and control group. SBP, systolic blood pressure; DBP, diastolic blood pressure.

Funnel plots and Egger's regression tests were used to evaluate for publication bias. The SBP funnel plot (Figure 3(a)) showed that the studies could not form a left-right symmetrical distribution, indicating publication bias in these studies. However, Egger's regression test of publication bias of SBP (*t* = −1.52, *P*=0.143＞0.05; Table 2) indicated no evidence of publication bias. The DBP funnel plot (Figure 3(b)) showed that a distribution with left and right symmetries could not be formed. Egger's regression test of publication bias for DBP (*t* = −2.22, *P*=0.037; Table 2) indicated significant publication bias. In addition, the funnel plot asymmetry may be due to the low-quality experiments with poor methodological design, imprecise data analysis, false-positive results, and small sample sizes.

### 3.3. Primary Outcomes

#### 3.3.1. SBP

**Figure 4:**
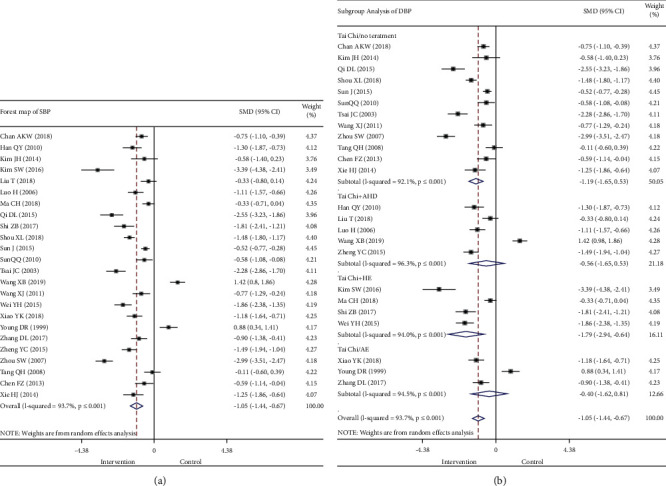
Forest map of all studies (a) and subgroup analysis (b) comparing SBP of the intervention group and the control group. SBP, systolic blood pressure.

The results of the meta-analysis of 24 studies that examined SBP indicators (Figure 4(a)) showed that Tai Chi lowered the SBP of participants compared with that of the control group participants (SMD: −1.05, 95% CI: −1.44, −0.67, *P* ≤ 0.001; Table 3). However, the meta-analysis results showed a statistically significant heterogeneity (*I*^*2*^ = 93.7%, *P* ≤ 0.001; Figure 4(a)). Therefore, the source of the heterogeneity should be further discussed.

The difference in the effect of Tai Chi on SBP in patients with high blood pressure may be affected by the intervention measures; therefore, the subgroup analysis was performed (Figure 4(b)) using a random response mode meta-analysis. The subgroup analysis showed that Tai Chi + AHD intervention had no statistical significance (*P*=0.314> 0.05; *I*^*2*^ = 96.3%), and the SMD after intervention with Tai Chi + HE (SMD −1.79, 95% CI −2.94 to −0.64, *P*=0.002; *I*^*2*^ = 94.0%) was higher than those with Tai Chi + AHD (SMD −0.56, 95% CI −1.65 to 0.53, *P*=0.314 > 0.05; *I*^*2*^ = 96.3%) and Tai Chi only (SMD −1.19, 95% CI −1.66 to −0.72, *P* ≤ 0.001; *I*^*2*^ = 92.1%) (Table 3). Three studies compared SBP of the Tai Chi group with that of the AE group, and results showed that there is no significant reduction of SBP in the Tai Chi group compared with the AE group (SMD −0.40, 95% CI −1.62 to 0.81, *P*=0.513 > 0.05; *I*^*2*^ = 94.5%).

Due to the differences in research characteristics, such as subject source, research quality, and intervention cycle, a multivariate meta-regression analysis was conducted with different research characteristics as covariables to explore the sources of heterogeneity. This analysis showed that the source of research objects (*t* = 0.44, *P*=0.666 > 0.05), research quality (*t* = 0.78, *P*=0.446 > 0.05), and intervention cycle (*t* = 0.44, *P*=0.667 > 0.05) as covariant quantities could not explain the interstitial heterogeneity (Supplementary Table 2).

Furthermore, an influence analysis of the individual studies was conducted (Supplementary Figure 1A). The remaining studies were combined after removing one study at a time to analyze the impact of individual studies on the combined results. The results of the analysis showed that studies by Zhou [20], Kim [8], and Wang [4] had the greatest impact. Significant heterogeneity (*I*^*2*^ > 50%) was still present, even after removing these data.

#### 3.3.2. DBP

**Figure 5:**
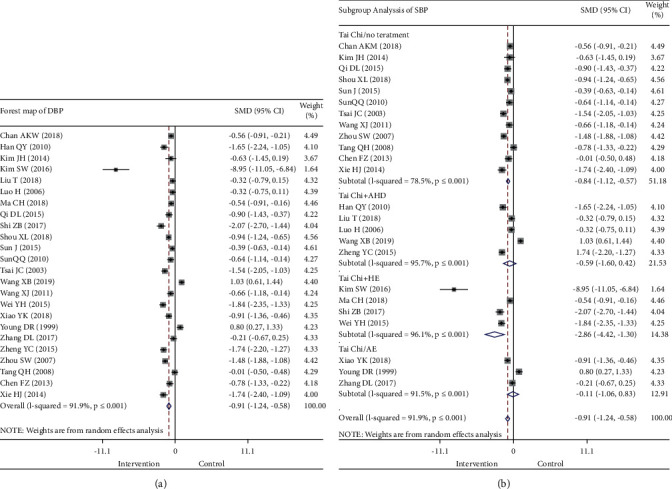
Forest map of all studies (a) and subgroup analysis (b) comparing DBP of the intervention group and the control group. DBP, diastolic blood pressure.

The meta-analysis performed on the 24 studies using DBP indicators found (Figure 5(a)) that Tai Chi exercise improved DBP in patients with hypertension (SMD −0.91, 95% CI −1.24 to −0.58, *P* ≤ 0.001; Table 3). Furthermore, the improvement in DBP was better in the intervention group than in the control group. Meta-analysis results showed significant heterogeneity, and the discrepancy was statistically significant (*I*^*2*^ = 91.9%, *P* ≤ 0.001; Figure 5(a)), which required further discussion on the sources of heterogeneity.

The difference in the effect of Tai Chi on DBP in patients with hypertension may have been affected by the intervention measures; therefore, a subgroup analysis was performed (Figure 5(b)) using the random response mode. The results showed that Tai Chi + AHD intervention had no statistical significance (*P*=0.253 > 0.05; *I*^*2*^ = 95.7%), in the Tai Chi group versus the AE group, there is no significant difference in reduction of DBP (SMD −0.11, 95% CI −1.06 to 0.83, *P*=0.812 > 0.05; *I*^*2*^ = 91.5%), and the SMD after intervention with Tai Chi + HE (SMD −2.86, 95% CI −4.42 to −1.30, *P* ≤ 0.001; *I*^*2*^ = 96.1%) was higher than that with Tai Chi + AHD (SMD −0.59, 95% CI −1.60 to 0.42, *P*=0.253 > 0.05; *I*^*2*^ = 95.7%) and Tai Chi only (SMD −0.84, 95% CI −1.12 to −0.57, *P* ≤ 0.001; *I*^*2*^ = 78.5%) (Table 3).

Multivariate meta-regression analysis was conducted using the research characteristics, such as source of research objects (*t* = −0.95, *P*=0.354 > 0.05), research quality (*t* = −0.74, *P*=0.468 > 0.05), and intervention cycle (*t* = −0.5, *P*=0.621 > 0.05), as covariables. The results indicated that the different research characteristics had no significant impact on interstudy heterogeneity (Supplementary Table 3).

Furthermore, an influence analysis of the individual studies was conducted (Supplementary Figure 1B). The remaining studies were combined after removing one study at a time to analyze the impact of individual studies on the combined results. The results of the analysis showed that the study by Kim [8] had the most significant impact. The remaining studies were combined after removing the data, indicating that there was still a high level of heterogeneity (*I*^*2*^ > 50%, *P* < 0.05).

### 3.4. Secondary Outcomes

#### 3.4.1. BMI

**Figure 6:**
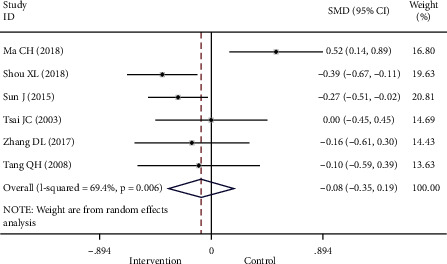
Forest map of studies comparing BMI of the intervention group and control group. BMI, body mass index.

Six studies [7, 10, 19, 22, 25, 26] reported BMI, and upon meta-analysis, no significant difference in BMI between the intervention and control groups was found (SMD −0.08, 95% CI −0.35 to −0.19, *P*=0.554; Table 3). The results of the meta-analysis showed that heterogeneity was significant (*I*^*2*^ = 69.4%, *P*=0.006; Figure 6).

#### 3.4.2. QOL

**Figure 7:**
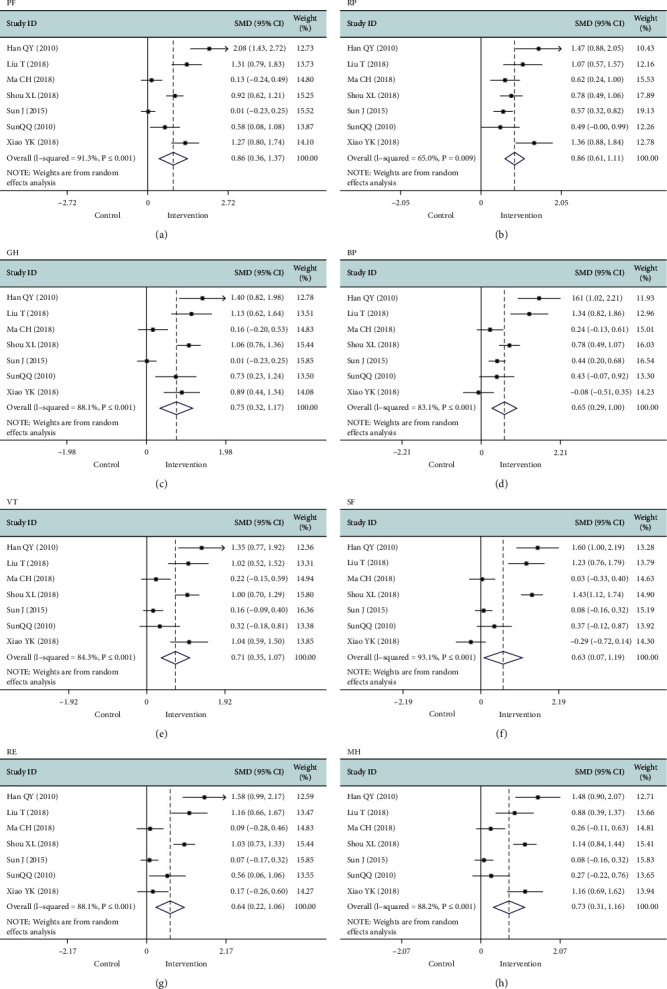
Forest map of the studies comparing PF (a), RP (b), GH (c), BP (d), VT (e), ST (f), RE (g), and MH (h) scores of the intervention group and control group. VT, vitality; SF, social functioning; RE, role-emotional; MH, mental health; PF, physical functioning; RP, role limitations due to physical health; BP, bodily pain; GH, general health perceptions.

Seven studies [10, 17, 18, 24–27] reported this outcome. The 36-Item Short Form Survey was used in all the studies; a higher score indicated a higher QOL. Upon conducting a meta-analysis, the following results were gathered: physical functioning (SMD 0.86, 95% CI 0.36 to 1.37, *P*=0.001; *I*^*2*^ = 91.3%), role-physical (SMD 0.86, 95% CI 0.61 to 1.11, *P* ≤ 0.001; *I*^*2*^ = 65%), general health (SMD 0.75, 95% CI 0.32 to 1.17, *P*=0.001; *I*^*2*^ = 88.1%), bodily pain (SMD 0.65, 95% CI 0.29 to 1.00, *P* ≤ 0.001; *I*^*2*^ = 83.1%), vitality (SMD 0.71, 95% CI 0.34 to 1.07, *P* ≤ 0.001; *I*^*2*^ = 84.3%), social functioning (SMD 0.63, 95% CI 0.07 to 1.19, *P*=0.027; *I*^*2*^ = 93.1%), role emotional (SMD 0.64, 95% CI 0.22 to 1.06, *P*=0.003; *I*^*2*^ = 88.1%), and mental health (SMD 0.73, 95% CI 0.31 to 1.16, *P*=0.001; *I*^*2*^ = 88.2%) (Table 3). So, the meta-analysis found that the intervention effect on QOL in patients with hypertension in the intervention group was better than that in the control group (Figure 7). In addition, the heterogeneity was significant, and the difference was statistically significant. We performed an influence analysis to explore potential sources of heterogeneity (Supplementary Figure 2), but the results did not change substantially.

## 4. Discussion

### 4.1. Summary of Findings

We included 24 RCTs (2,095 patients) in this meta-analysis, with two [4, 23] reporting Tai Chi's lack of antihypertensive effects. The meta-analysis found that the improvement in SBP and DBP among patients with hypertension in the intervention group was better than that seen in the control group, and the former also had improved QOL. However, Tai Chi intervention did not improve BMI.

### 4.2. Added Value to Previous Meta-Analysis on the Same Topic

There have been six previous systematic reviews [31–36] examining the effects of Tai Chi on hypertension. The reasons for our update were as follows: first, we focused on the influence of duration of exercise on the results. According to a meta-analysis by Zhang [32], if the exercise duration was less than three weeks, Tai Chi would not significantly reduce DBP. As such, we only included studies that had at least three weeks of intervention. Second, there was an issue of whether the effects of Tai Chi on blood pressure was applicable to different ethnicities. At the same time, we found that all patients included in the previous meta-analysis were from China. Therefore, we conducted a comprehensive search and found two articles from South Korea [8, 13] and one from Baltimore, USA [23]. We only included RCTs to avoid having incorrect results due to the inclusion of non-RCTs or incomplete data. Third, we found that in some studies, the medications, combined with Tai Chi, were not gradually reduced despite the increase in Tai Chi exercise; this could profoundly influence Tai Chi's effect on blood pressure. However, the previous meta-analyses were not mindful of this, so we conducted a subgroup analysis of different interventions. Fourth, most of the studies only performed a meta-analysis using blood pressure as an outcome, but we found that other secondary indicators, such as QOL, may explain the mechanism underlying Tai Chi's effect on blood pressure. Therefore, secondary indexes, such as BMI, WC, and QOL, were added to the data collection.

### 4.3. Interpretation of the Results

#### 4.3.1. SBP and DBP

The meta-analysis results showed that Tai Chi intervention could significantly improve SBP and DBP in patients with hypertension. Tai Chi reduces blood pressure through various means. First, during Tai Chi exercise, the amount of sodium lost may exceed the regular intake level, leading to an improvement in blood pressure [22]. Second, after exercise, Tai Chi practitioners had higher plasma levels of NO metabolites than sedentary and sedentary participants did [37]. Furthermore, as an endothelium-dependent vasodilator, NO plays a significant role in regulating vascular tension [38]. NO can also reduce skin vascular resistance, thereby reducing hypertension. Third, NLRP3 inflammasome damages endothelial cells, and exercise reduces the expression of NLRP3 inflammasome components, thereby lowering blood pressure [39, 40]. Fourth, performing Tai Chi can cultivate the mind, edify sentiments, make people broad-minded, open-minded, and optimistic, and eliminate the effects of bad mood on the nervous system caused by changes in blood pressure [41].

We performed a subgroup analysis of different interventions. Tai Chi intervention alone was used in twelve of the included articles, and the results showed that Tai Chi intervention alone could dramatically improve SBP and DBP in patients with essential hypertension. However, the subgroup analysis results of Tai Chi combined with conventional Western medicines showed no statistic significant changes, which do not automatically indicate that Tai Chi has no antihypertensive effect. This result may have been due to the simultaneous use of Tai Chi and drugs, complicating the study and making it difficult to distinguish the effects of the two interventions. If the drugs were gradually increased or decreased after the Tai Chi intervention and the changes in blood pressure were recorded simultaneously, the effect of Tai Chi intervention on prehypertension could be understood, and the complexity of the intervention could be reduced to obtain more accurate results. There are two methods to reduce this complexity in the intervention and obtain more accurate results. First, after the Tai Chi intervention, the gradual increase or decrease in the dose of drugs should be noted while observing the changes in blood pressure. Second, we can study the intervention effect of Tai Chi on prehypertension, that is, the intervention of people with high normal blood pressure who has not taken medicine, so as to observe the influence of Tai Chi on blood pressure. In addition, the subgroup analysis of Tai Chi combined with HE showed that the antihypertensive effect was greater than that with Tai Chi intervention alone, possibly because HE includes dietary, exercise, lifestyle changes, and stress reduction, all of which may enhance the effect of blood pressure reduction.

The subgroup analysis showed that Tai Chi presents no significant antihypertensive effects on SBP and DBP compared with AE. At present, professional organizations recommend AE as the primary mode of exercise to prevent and treat hypertension [42]. However, very few adults with high blood pressure follow this recommendation [43]. In the 60–69 years age group, a reduction of 10 mmHg in SBP and 5 mmHg in DBP was associated with a 22% and 41% decrease in the risk of heart disease and stroke, respectively [44]. Patients of this age are more suited to do Tai Chi than AE for physical reasons. Therefore, it can be used as a substitute for under certain conditions, such as when people are unable or unwilling to perform AE.

#### 4.3.2. QOL

The results of the meta-analysis showed that Tai Chi could improve the QOL of patients with hypertension as it strengthens the body and improves the mind. With improved mental and physical control, people's motivation to continue to exercise increases, as does their overall well-being [45]. Increased circulating levels of *β*-endorphins, which are associated with chronic pain, are potential biomarkers of endogenous opioid analgesic power [46]. Compared to light exercise, Tai Chi reduces plasma *β*-endorphin levels more efficiently, thereby reducing chronic pain [47]. It can also help patients overcome their fear of pain and improve their psychosocial health status and confidence [48].

Elderly individuals are prone to falls, one of the main risk factors for poor QOL. The probability of falling increases with age. Worldwide, the annual incidence of falls is around 30–40% among people over 65 years of age but increases to 50% among people 80 years of age and over [49]. Tai Chi exercise for elderly individuals helps prevent falls, with increased frequency of Tai Chi exercise being associated with reduced frequency of falls [50]. Furthermore, people with fibromyalgia may also benefit from Tai Chi exercise [51]. Overall, by regulating their body and mind, Tai Chi can improve people's mood, pain, and general health, thus improving their QOL.

### 4.4. Limitation

This study had the following limitations: (1) the heterogeneity was high among selected trials. There are some additional unidentified moderators existing in included studies which may cause considerable heterogeneity, such as the intensity of Tai Chi practice, the intervention duration, and the frequency of Tai Chi training. (2) Only two of the 24 included studies mentioned blinding methods. For evaluators, which may lead to deception and performance bias. (3) The number of samples in one article was negligible. (4) The studies also did not adequately report the patients' medical adherence and the teaching ability of the coach. Therefore, we should maintain a conservative attitude towards interpretation of the results that imply meaningful reduction of hypertension caused by Tai Chi.

### 4.5. Conclusion

The results show that Tai Chi exercise can effectively improve SBP, DBP, and QOL among patients with hypertension. As such, it should be promoted as a safe and effective adjuvant therapy for hypertension. The results of this study can be used as a theoretical basis for guiding clinical practice. Nevertheless, because of the substantial interstudy heterogeneity and the influence of publication bias, these findings still need to be verified by a rigorously designed study with a large sample size.

## Figures and Tables

**Table 1:** Basic characteristics of the included trials.

Study	Year	Sample (T/C)	Age	Intervention	Control	Duration	Frequency	Outcome
Wang and Ye	2019	50/50	60–80	Tai Chi + AHD	AHD	3 M	3 times per week for 40–60 min	①
Chan et al.	2018	69/62	30–91	Tai Chi	No treatment	9 M	At least 5 times per week for 30 min	①
Liu et al.	2018	35/35	I:62.4 ± 2.4 C:63.1 ± 2.1	Tai Chi + AHD	AHD	6 M	Once a day for 40–60 min	①⑤
Ma et al.	2018	55/58	60 or over	Tai Chi + HE	HE	24 W	3–5 times per week for at least 60 min	①②③⑤
Shou et al.	2018	98/100	18–60	Tai Chi	No treatment	3 M	Once a day for 20–30 min	①②⑤
Xiao et al.	2018	42/42	I:60.2 ± 4.6 C:60.5 ± 4.9	Tai Chi	AE	3 M	5 times per week for 60 min	①④⑤
Shi and Mao	2017	30/30	30–55	Tai Chi + HE	HE	3 M	4–5 times per week for 30 min	①
Zhang	2017	36/37	60–80	Tai Chi	AE	12 W	3 times per week for 60 min	①②
Kim et al.	2016	20/20	I:73.70 ± 1.69 C:73.20 ± 1.61	Tai Chi + HE	HE	24 W	3–5 times per week for 45 min	①
Qi et al.	2015	30/30	NA	Tai Chi	No treatment	12 W	5 times per week for 60 min	①
Sun and Buys	2015	136/130	45–80	Tai Chi	No treatment	1 Y	5 h per week	①②③⑤
Wei et al.	2015	42/42	I:72 ± 5.56 C:70 ± 6.08	Tai Chi + HE	HE	1 Y	Once a day for 30–45 min	①
Zheng et al.	2015	49/49	I:54.71 ± 5.43 C:55.77 ± 6.24	Tai Chi + AHD	AHD	12 W	4–8 times per week for 40–60 min	①
Kim et al.	2014	12/12	I:78.8 ± 5.4 C:76.2 ± 4.6	Tai Chi	No treatment	12 W	3 times per week for 120 min	①
Xie and Bai	2014	25/25	60–70	Tai Chi	No treatment	12 W	5 times per week for 1 h	①④
Chen and Lu	2013	50/18	30–82	Tai Chi	No treatment	12 W	6 times per week for 30 min	①
Wang et al.	2011	30/30	50–70	Tai Chi	No treatment	16 W	5 times per week for 60 min	①
Han et al.	2010	30/28	62.21 ± 10.51	Tai Chi + AHD	AHD	5 Y	Once a day for 45–60 min	①⑤
Sun	2010	32/32	40–70	Tai Chi	No treatment	3 M	6 times per week for 90 min	①⑤
Tang	2008	32/32	60–70	Tai Chi	No treatment	12 M	5–7 times per week for 1 h	①②③
Zhou	2007	60/60	I:52.3 ± 10.7; C:53.4 ± 11.2	Tai Chi	No treatment	12 W	6 times per week for 60 min	①
Luo	2006	44/42	I:44.75 ± 12.10; C:44.86 ± 13.05	Tai Chi + AHD	AHD	6 M	Once a day for 45 min	①
Tsai et al.	2003	37/39	I:51.6 ± 16.3 C:50.5 ± 9.8	Tai Chi	No treatment	12 W	3 times per week for 50 min	①②
Young et al.	1999	30/30	60–80	Tai Chi	AE	12 W	4-5 times per week for 30–45 min	①

AHD, antihypertensive drugs; HE, health education; AE, aerobic exercise; SBP, systolic blood pressure; DBP, diastolic blood pressure; BMI, body mass index; WC, waist circumference; NO, nitric oxide content; I, intervention group; C, control group. ①, SBP and DBP; ②, BMI; ③, WC; ④, NO; ⑤, quality of life.

**Table 2:** Literature publication bias test.

	Coefficient	Standard error	*t*	*P*	95% CI
SBP	−4.181	2.756	−1.52	0.143	−9.896, 1.534
DBP	−4.755	2.144	−2.22	0.037	−9.202, −0.308

SBP, systolic blood pressure; DBP, diastolic blood pressure; CI, confidence intervals.

**Table 3:** Results of meta-analysis and subgroup analysis.

		SBP	DBP	BMI	QOL							
					PF	RP	GH	BP	VT	SF	RE	MH
Invention group vs. control group	*I*^2^ (%)	93.7	91.9	69.4	91.3	65	88.1	83.1	84.3	93.1	88.1	88.2
	SMD	−1.05	−0.91	−0.08	0.86	0.86	0.75	0.65	0.71	0.63	0.64	0.73
	95% CI	(−1.44, −0.67)	(−1.24, −0.58)	(−0.35, 0.19)	(0.36, 1.37)	(0.61, 1.11)	(0.32, 1.17)	(0.29, 1.00)	(0.34, 1.07)	(0.07, 1.19)	(0.22, 1.06)	(0.31, 1.16)
	*Z*	5.39	5.35	0.59	3.38	6.67	3.44	3.56	3.75	2.21	2.96	3.36
	*P*	≤0.001	≤0.001	0.554	0.001	≤0.001	0.001	≤0.001	≤0.001	0.027	0.003	0.001

Tai Chi group vs. no treatment	*I*^2^ (%)	92.1	78.5	NA	NA	NA	NA	NA	NA	NA	NA	NA
	SMD	−1.19	−0.84	NA	NA	NA	NA	NA	NA	NA	NA	NA
	95% CI	(−1.66, −0.72)	(−1.12 −0.57)	NA	NA	NA	NA	NA	NA	NA	NA	NA
	*Z*	4.98	5.99	NA	NA	NA	NA	NA	NA	NA	NA	NA
	*P*	≤0.001	≤0.001	NA	NA	NA	NA	NA	NA	NA	NA	NA

Tai Chi + AHD group vs. control group	*I*^2^ (%)	96.3	95.7	NA	NA	NA	NA	NA	NA	NA	NA	NA
	SMD	−0.56	−0.59	NA	NA	NA	NA	NA	NA	NA	NA	NA
	95% CI	(−1.65, 0.53)	(−1.60, 0.42)	NA	NA	NA	NA	NA	NA	NA	NA	NA
	*Z*	1.01	1.14	NA	NA	NA	NA	NA	NA	NA	NA	NA
	*P*	0.314	0.253	NA	NA	NA	NA	NA	NA	NA	NA	NA

Tai Chi + HE group vs. control group	*I*^2^ (%)	94	96.1	NA	NA	NA	NA	NA	NA	NA	NA	NA
	SMD	−1.79	−2.86	NA	NA	NA	NA	NA	NA	NA	NA	NA
	95% CI	(−2.94, −0.64)	(−4.42, −1.30)	NA	NA	NA	NA	NA	NA	NA	NA	NA
	*Z*	3.06	3.59	NA	NA	NA	NA	NA	NA	NA	NA	NA
	*P*	0.002	≤0.001	NA	NA	NA	NA	NA	NA	NA	NA	NA

Tai Chi group vs. AE group	*I*^2^ (%)	94.5	91.5	NA	NA	NA	NA	NA	NA	NA	NA	NA
	SMD	−0.4	−0.11	NA	NA	NA	NA	NA	NA	NA	NA	NA
	95%CI	(−1.62, 0.81)	(−1.06, 0.83)	NA	NA	NA	NA	NA	NA	NA	NA	NA
	*Z*	0.65	0.24	NA	NA	NA	NA	NA	NA	NA	NA	NA
	*P*	0.513	≤0.001	NA	NA	NA	NA	NA	NA	NA	NA	NA

SBP, systolic blood pressure; DBP, diastolic blood pressure; BMI, body mass index; PF, physical functioning; RP, role-physical; GH, general health; BP, bodily pain; VT, vitality; SF, social functioning; RE, role-emotional; MH, mental health; AHD, antihypertensive drugs; HE, health education; SMD, standard mean.

## Data Availability

The manuscript is a meta-analysis. With a time limit of December 5, 2020, we searched for RCTs in five English and two Chinese databases (Web of Science, PubMed, Korea Citation Index, EMBASE, the Cochrane Library, China National Knowledge Infrastructure, and Wanfang Data). We searched for terms related to Tai Chi and hypertension. Overall, the data of 2,095 patients from 24 RCTs (Young et al., 1999; Tsai et al., 2003; Luo, 2006; Zhou, 2007; Tang, 2008; Qiao-ying et al., 2010; Sun, 2010; Wang et al., 2011; Chen and Lu, 2013; Kim, 2014; Xie and Bai, 2014; Qi et al., 2015; Sun and Buys, 2015; Wei et al., 2015; Zheng et al., 2015; Kim et al., 2016; Shi and Miao, 2017; Zhang, 2017; Chan et al., 2018; Liu et al., 2018; Ma et al., 2018; Shou et al., 2018; Xiao, 2018; and Wang and Ye, 2019) were obtained, as given in Table 1 and Supplementary Table 1 in the manuscript.

## Supplementary Materials

Supplementary Materials. Supplementary Table 1. The difference in values before and after intervention. SBP, systolic blood pressure; DBP, diastolic blood pressure; BMI, body mass index; WC, waist circumference; NO, nitric oxide content; QOL, quality of life; PF, physical functioning; RP, role limitations due to physical health; BP, bodily pain; GH, general health perceptions; VT, vitality; SF, social functioning; RE, role limitations due to emotional problems; MH, mental health; I, intervention group; C, control group. Supplementary Table 2. Multivariate meta-regression analysis of studies evaluating systolic blood pressure in patients with hypertension doing Tai Chi. Coef, coefficient; Std.Err, standard error; CI, confidence intervals. Supplementary Table 3. Multivariate meta-regression analysis of studies evaluating diastolic blood pressure in patients with hypertension doing Tai Chi. Coef, coefficient; Std.Err, standard error; CI, confidence intervals. Supplementary Figure 1. Influence analysis of studies assessing SBP (A) and DBP (B) in patients with hypertension by Tai Chi. SBP, systolic blood pressure; DBP, diastolic blood pressure. Supplementary Figure 2. Influence analysis of studies assessing PF (A), RP (B), GH (C), BP (D), VT (E), ST (F), RE (G), and MH (H) scores in patients with hypertension by Tai Chi. VT, vitality; SF, social functioning; RE, role limitations due to emotional problems; MH, mental health; PF, physical functioning; RP, role limitations due to physical health; BP, bodily pain; GH, general health perceptions.
