# Supplementary material for: Tai Chi as a Therapy of Traditional Chinese Medicine on Reducing Blood Pressure: A Systematic Review of Randomized Controlled Trials
Source: Evid Based Complement Alternat Med. 2021 Sep 4;2021:4094325. doi: 10.1155/2021/4094325 (PMC8437614; doi:10.1155/2021/4094325)
Supplement: Supplementary Materials. — Supplementary Table 1. The difference in values before and after intervention. SBP, systolic blood pressure; DBP, diastolic blood pressure; BMI, body mass index; WC, waist circumference; NO, nitric oxide content; QOL, quality of life; PF, physical functioning; RP, role limitations due to physical health; BP, bodily pain; GH, general health perceptions; VT, vitality; SF, social functioning; RE, role limitations due to emotional problems; MH, mental health; I, intervention group; C, control group. Supplementary Table 2. Multivariate meta-regression analysis of studies evaluating systolic blood pressure in patients with hypertension doing Tai Chi. Coef, coefficient; Std.Err, standard error; CI, confidence intervals. Supplementary Table 3. Multivariate meta-regression analysis of studies evaluating diastolic blood pressure in patients with hypertension doing Tai Chi. Coef, coefficient; Std.Err, standard error; CI, confidence intervals. Supplementary Figure 1. Influence analysis of studies assessing SBP (A) and DBP (B) in patients with hypertension by Tai Chi. SBP, systolic blood pressure; DBP, diastolic blood pressure. Supplementary Figure 2. Influence analysis of studies assessing PF (A), RP (B), GH (C), BP (D), VT (E), ST (F), RE (G), and MH (H) scores in patients with hypertension by Tai Chi. VT, vitality; SF, social functioning; RE, role limitations due to emotional problems; MH, mental health; PF, physical functioning; RP, role limitations due to physical health; BP, bodily pain; GH, general health perceptions. [file 4094325.f1.docx]

**Supplementary materials**

**Supplementary Table 1.** The difference in values before and after the intervention.

| **Study** | **Blood pressure** | | **BMI** | **WC** | **NO** | **QOL** | | | | | | | |
| --- | --- | --- | --- | --- | --- | --- | --- | --- | --- | --- | --- | --- | --- |
|  | **I** | **C** |  |  |  | **PF** | **RP** | **GH** | **BP** | **VT** | **SF** | **RE** | **MH** |
| Wang XB | SBP: -26.28; DBP: -17.04 | SBP: -40.66; DBP: -26.4 | NA | NA | NA | NA | NA | NA | NA | NA | NA | NA | NA |
| Ma CH | SBP: -4.69; DBP: -6.21 | SBP: 1.55; DBP: -1.56 | I: -1.78; C: -3.51 | I: -2.65; C: -1.35 | NA | I: 2.15; C: 0.32 | I: 11.6; C: -1.19 | I: 4.72; C: 1.08 | I: 5.28; C: 0.6 | I: 6.17; C: 1.47 | I: 0.91; C: 0.14 | I: 3.40; C: 1.54 | I: 6.39; C: 1.49 |
| Chan AKW | SBP: -12.81; DBP: -5.28 | SBP: -0.23; DBP: 1.02 | NA | NA | NA | NA | NA | NA | NA | NA | NA | NA | NA |
| Shou XL | SBP: -12.74; DBP: -6.92 | SBP: 0.01; DBP: 0.89 | I: -0.88; C: -0.04 | NA | NA | I:12.3 C:4.8 | I: 11.6; C: 0.5 | I: 16.5; C: 3.1 | I: 15.2; C: 4.8 | I: 8.7; C: -1.8 | I: 19.5; C: 0.7 | I: 16.2; C: 1.0 | I: 15.9; C: 1.3 |
| Liu T | SBP: -22.07; DBP: -11.40 | SBP: -17.29; DBP: -8.44 | NA | NA | NA | I:13.0 C:2.2 | I: 16.7; C: 1.1 | I: 16.0; C: 1.4 | I: 15.6; C: 0.3 | I: 9.6; C: -1.0 | I: 18.2; C: 1.3 | I: 16.4; C: 1.3 | I: 13.8; C: 2.1 |
| Xiao YK | SBP: -26.1; DBP: -12.9 | SBP: -14.6; DBP: -4.3 | NA | NA | I: 22.8; C: 5.1 | I: 27.9; C: 15.6 | I: 23.4; C: 12.3 | I: 21.9; C: 14.5 | I: 1.7; C: 2.4 | I: 24.0; C: 14.1 | I: 2.3; C: 4.8 | I: 2.9; C: 1.4 | I: 24.6; C: 14.7 |
| Shi ZB | SBP: -10.43; DBP: -6.90 | SBP: -2.33; DBP: 2.34 | NA | NA | NA | NA | NA | NA | NA | NA | NA | NA | NA |
| Zhang DL | SBP: -10.75; DBP: -2.53 | SBP: -1.76; DBP: -0.84 | I: -0.1; C: 0.4 | NA | NA | NA | NA | NA | NA | NA | NA | NA | NA |
| Kim SW | SBP: -6.9; DBP: -15.5 | SBP: 0.1; DBP: -0.95 | NA | NA | NA | NA | NA | NA | NA | NA | NA | NA | NA |
| Sun J | SBP: -10.43; DBP: -6.90 | SBP: -2.33; DBP: -2.34 | I: -1.13; C: -0.34 | I: -0.33; C: -0.33 | NA | I: -0.69; C: -0.92 | I: 11.31; C: 0.79 | I: 4.01; C: 3.66 | I: 11.35; C: 1.79 | I: 9.77; C: 6.22 | I: 2.54; C: 1.18 | I: 3.54; C: 2.18 | I: 3.87; C: 2.37 |
| Qi DL | SBP: -25.5; DBP: -9.06 | SBP: -0.7; DBP: -0.8 | NA | NA | NA | NA | NA | NA | NA | NA | NA | NA | NA |
| Zheng YC | SBP: -40.03; DBP: -25.79 | SBP: -24.98; DBP: -14.69 | NA | NA | NA | NA | NA | NA | NA | NA | NA | NA | NA |
| Wei YH | SBP: -16.8; DBP: -13.1 | SBP: 7; DBP: 5.1 | NA | NA | NA | NA | NA | NA | NA | NA | NA | NA | NA |
| Kim JH | SBP: -0.83; DBP: -1.34 | SBP: 0.5; DBP: 0.17 | NA | NA | NA | NA | NA | NA | NA | NA | NA | NA | NA |
| Xie HJ | SBP: -12.66; DBP: -14.19 | SBP: -1.16; DBP: 1.23 | NA | NA | I: 7.69; C: -0.66 | NA | NA | NA | NA | NA | NA | NA | NA |
| Chen FZ | SBP: -12.9; DBP: -7.5 | SBP: -1.3; DBP: -1.8 | NA | NA | NA | NA | NA | NA | NA | NA | NA | NA | NA |
| Wang XJ | SBP: -13.9; DBP: -8.13 | SBP: -0.93; DBP: -0.93 | NA | NA | NA | NA | NA | NA | NA | NA | NA | NA | NA |
| Sun QQ | SBP: -13.90; DBP: -8.13 | SBP: -0.93; DBP: -0.93 | NA | NA | NA | I: 6.09; C: -1.1 | I: 21.09; C: 2.34 | I: 14.72; C: 1.22 | I: 7.69; C: 1.00 | I: 7.18; C: 1.88 | I: 7.64; C: 1.39 | I: 16.67; C: -5.21 | I: 3.18; C: -0.87 |
| Han QY | SBP: -35.2; DBP: -26.8 | SBP: -24.7; DBP: -13.1 | NA | NA | NA | I: 12.2; C: -9.9 | I: 12.8; C: -6.9 | I: 14.8; C: -4.0 | I: 13.5; C: -3.7 | I: 8.0; C: -5.1 | I: 18.5; C: -3.3 | I: 18.4; C: -2.6 | I: 17.9; C: -1.9 |
| Tang QH | SBP: -10.29; DBP: -0.94 | SBP: 3.09; DBP: -0.19 | I: -1.94; C: -0.36 | I: -4.18; C: 0.15 | NA | NA | NA | NA | NA | NA | NA | NA | NA |
| Zhou SW | SBP: -24.2; DBP: -11.0 | SBP: -6.0; DBP: -4.1 | NA | NA | NA | NA | NA | NA | NA | NA | NA | NA | NA |
| Luo H | SBP: -33.14; DBP: -7.91 | SBP: -20.21; DBP: -5.22 | NA | NA | NA | NA | NA | NA | NA | NA | NA | NA | NA |
| Tsai JC | SBP: -15.6; DBP: -8.8 | SBP: 6.4; DBP: 3.4 | I: -0.2; C: -0.2 | NA | NA | NA | NA | NA | NA | NA | NA | NA | NA |
| Young DR | SBP: -7.0; DBP: -2.4 | SBP: -8.4; DBP: -3.2 | NA | NA | NA | NA | NA | NA | NA | NA | NA | NA | NA |

SBP, systolic blood pressure; DBP, diastolic blood pressure; BMI, body mass index; WC, waist circumference; NO, nitric oxide content; QOL, quality of life;

PF, physical functioning; RP, role limitations due to physical health; BP, bodily pain; GH, general health perceptions; VT, vitality; SF, social functioning;RE,

role limitations due to emotional problems; MH, mental health; I, intervention group; C, control group.

**Supplementary Table 2.** Multivariate Meta Regression Analysis of Studies

valuating Systolic Blood Pressure in Patients with Hypertension doing Tai Chi.

|  | **Coef** | **Std. Err** | **t** | **P** | **[95%CI]** |
| --- | --- | --- | --- | --- | --- |
| Research objects | 0.333 | 0.759 | 0.44 | 0.666 | -1.251,1.917 |
| Research quality | -0.266 | 0.342 | -0.78 | 0.446 | -0.978,0.447 |
| Intervention cycle | -0.145 | 0.331 | -0.44 | 0.667 | -0.835,0.546 |

Coef, coefficient; Std.Err, Standard error; CI, confidence intervals.

**Supplementary Table 3.** Multivariate Meta Regression Analysis of Studies

Evaluating Diastolic Blood Pressure in Patients with Hypertension doing Tai Chi.

|  | **Coef** | **Std.Err** | **t** | **P** | **[95%CI]** |
| --- | --- | --- | --- | --- | --- |
| Research objects | -1.034 | 1.089 | -0.95 | 0.354 | -3.306,1.238 |
| Research quality | -0.353 | 0.478 | -0.74 | 0.468 | -1.349,0.643 |
| Intervention cycle | -0.232 | 0.462 | -0.5 | 0.621 | -1.196,0.732 |

Coef, coefficient; Std.Err, Standard error; CI, confidence intervals.


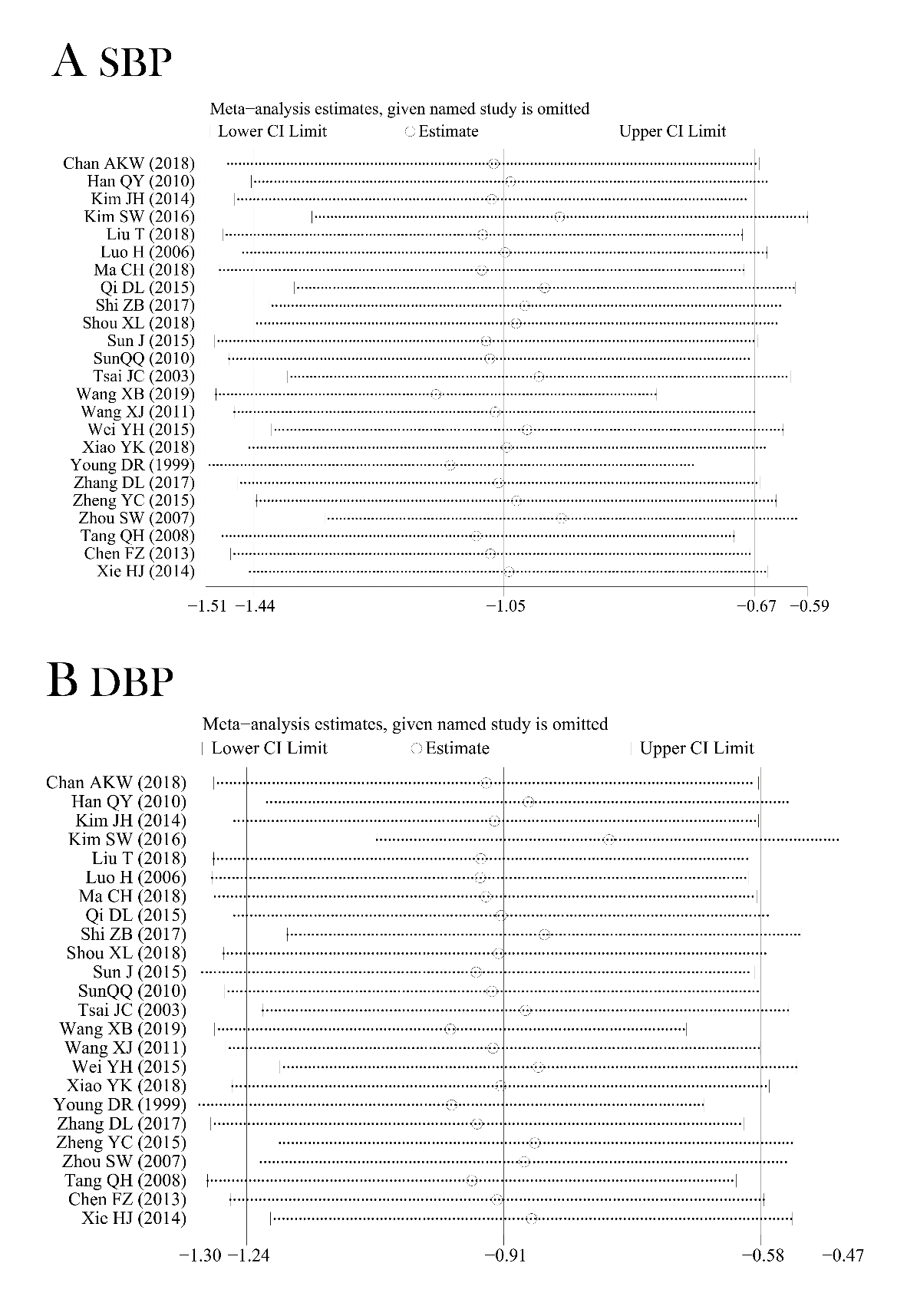


**Supplementary Figure 1.** Influence Analysis of Studies Assessing SBP (A) and DBP (B) in Patients with Hypertension by Tai Chi.( SBP, systolic blood pressure; DBP, diastolic blood pressure。）


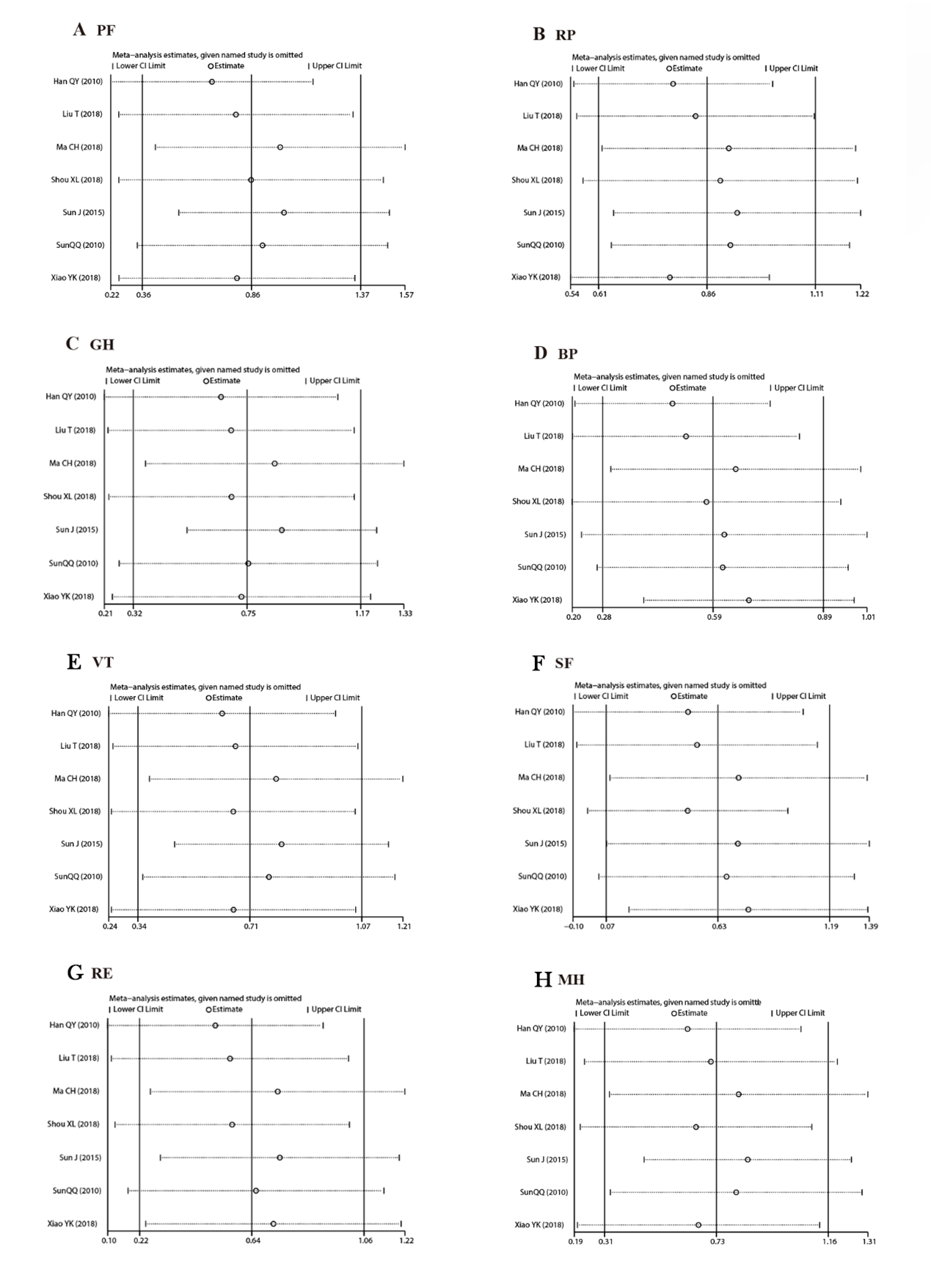
**Supplementary Figure 2.** Influence Analysis of Studies Assessing PF (A), RP (B), GH (C), and BP (D), VT (E), SF (F), RE (G) and MH (H) in Patients with Hypertension by Tai Chi. (PF, physical functioning; RP, role limitations due to physical health; BP, bodily pain; GH, general health perceptions; VT, vitality; SF, social functioning; RE, role limitations due to emotional problems; MH, mental health.)
